# Effect of Postmortem Degradation on the Preservation of Viral Particles and Rabies Antigens in Mice Brains. Light and Electron Microscopic Study

**DOI:** 10.3390/v12090938

**Published:** 2020-08-26

**Authors:** Jeison Monroy-Gómez, Gerardo Santamaría, Ladys Sarmiento, Orlando Torres-Fernández

**Affiliations:** 1 Grupo de Morfología Celular, Instituto Nacional de Salud (INS), 111321 Bogotá, D.C., Colombia; biojulmilger@gmail.com (G.S.); lsarmiento@ins.gov.co (L.S.); 2 Rehabilitation School of Colombia, Institución Universitaria Escuela Colombiana de Rehabilitación, 110121 Bogotá, D.C., Colombia

**Keywords:** Rabies, rabies virus, postmortem degradation, Negri body, immunohistochemistry, electron microscopy

## Abstract

Rabies diagnosis is mainly made on fresh brain tissue postmortem by means of the direct immunofluorescence test. However, in some cases, it is not possible to use this technique, given that the affected nervous tissue goes through a postmortem degradation process, due to problems in the handling and transport of the samples. For this reason, the preservation in time of the rabies virus inclusions was assessed, as well as the immunoreactivity and the ultrastructure of viral particles in tissue with postmortem degradation. Brains of mice inoculated with rabies virus and control mice were processed for conventional histology, immunohistochemistry, electron microscopy, and immunoelectron microscopy in different postmortem times. In the processed tissues for hematoxylin and eosin, the presence of eosinophilic inclusions was not observed beyond 12 h postmortem. Surprisingly, the immunoreactivity of the viral antigens increased with time, at least until 30 h postmortem. It was possible to easily recognize the viral particles by means of conventional electron microscopy until 12 h postmortem. Immunoelectron microscopy allowed us to identify the presence of viral antigens disseminated in the neuronal cytoplasm until 30 h postmortem, but immunoreactive viral particles were not observed. The rabies infection did not cause histological or ultrastructural alterations different from those in the control group as a result of the postmortem degradation. In conclusion, the immunohistochemistry is a reliable test for rabies diagnosis in samples with postmortem degradation and that have been fixed with aldehydes.

## 1. Introduction

Rabies is a viral infection affecting the central nervous system of mammals. Rabies virus is an RNA virus from the family *Rhabdoviridae* and the genus *Lyssavirus*. Every year, 55,000 people die in the world because of this disease. A timely and reliable diagnosis of rabies is part of the recommendations of the OIE (World Organization for Animal Health) and the WHO (World Health Organization) to prevent and control the disease [1]. The gold standard for rabies diagnosis is the direct immunofluorescence test, which identifies proteins from the nucleocapsid of the virus in fresh tissue [1,2,3,4]. However, test reliability depends on the preservation of the sample. Postmortem degradation in brain tissue can happen for diverse reasons, producing alterations in antigenic epitopes, as well as substances derived from tissue decomposition, which cause false negatives or false positives [5,6]. There are several studies on the effects of postmortem degradation in brain tissue for rabies diagnosis when it is made through direct immunofluorescence and molecular techniques [6,7,8].

Usually, human tissues are only available for histological studies after several hours postmortem. Frequently, the brains obtained by necropsy are formaldehyde-fixed, because a rabies diagnosis is not suspected, or because there are problems to find a safe and timely transport for the fresh brains. Inmunohistochemistry is a reliable technique for rabies diagnosis in nervous tissue fixed in aldehydes [9,10,11,12,13]. In addition, the material fixed in aldehydes allows performing electron microscopy studies that can contribute to diagnosis and provide additional information on the effects of the infection in the nervous tissue [14,15,16]. Nonetheless, we did not find published information on the effect of postmortem degradation of brain tissue in the immunohistochemistry and ultrastructural diagnosis of rabies. This work was carried out with the purpose of assessing the preservation of the rabies virus inclusions (sometimes called Negri bodies) [9], antigens immunoreactivity, and the viral particles ultrastructure on mice brains inoculated with the rabies virus and fixed in paraformaldehyde in diverse postmortem times.

## 2. Material and Methods

### 2.1. Ethics Statement

This research on rabies virus in mouse and the protocol for use of animals was discussed and approved among the staff members of Ethics Committee of the Instituto Nacional de Salud (INS), Colombia; all the procedures were carried out in accordance with the approved guidelines (Minutes No. 8, October 13, 2011).

### 2.2. Animal Handling, Virus Inoculation, and Collection of the Infected Tissue

The rabies virus used to carry out this study was the wild type, of canine origin, supplied by the Virology Laboratory of the Instituto Nacional de Salud (INS), Colombia. The animals used for the experiment were 21 female 28-days-old ICR-CD1 (Institute of Cancer Research) (28 gr weigh) mice, which were kept in a high-security room of the vivarium (BSL-2) of INS in optimal environmental and nutritional conditions (*Ad libitum*), according to the ethical and legal norms demanded for research with laboratory animals. Fourteen mice were inoculated via intracerebral, each of them with 0.03 mL of viral dilution (LD_50_ = 10^6.6^) using syringes with fine needles (No. 27). As controls, six mice of the same age were used and inoculated with a vehicle solution without virus (2% normal horse serum dissolved in water plus antibiotics: 200 IU/mL of penicillin and 4 mg/mL of streptomycin). The animals were kept under observation during the following days, until 120 h postinoculation, when they developed advanced symptoms of the disease (lying at the bottom of the cage without any locomotor activity and with low body temperature at contact). The sacrifice of the animals started at that moment as described below.

Two infected animals and one control were sacrificed at 0 h postmortem. Initially, they were anesthetized with 0.2 mL of chloral hydrate at 30% (Merck, Darmstadt, Germany) administered via intraperitoneal (350 mg/Kg). Immediately after, they were fixed by intracardiac perfusion. First, the phosphate buffered saline (PBS) with a pH of 7.3 circulated for 3 min (approximately 30 mL), and then a paraformaldehyde solution at 4% for 10 min (approximately 100 mL). Once the perfusion was over, the brains were extracted from each mouse, and they were preserved in paraformaldehyde at 4%. The rest of the animals were sacrificed in a CO_2_ chamber and beheaded to bleed them out. Heads were left at room temperature (approx. 22 °C). The extraction of the entire brain was made at different postmortem times (0, 6, 12, 18, 24, and 30 h), at each time interval the brains from two infected animals and their corresponding control were extracted. Immediately after, they were submerged in PFA at 4% and were kept at 4 °C for a minimum time of 48 h, before continuing with the other procedures.

### 2.3. Histological and Immunohistochemical Processing

From each encephalon, infected and control, brain slices were oriented in a coronal plane and cerebellum slices in a sagittal plane. Those of about 0.5 cm thick were cut under a stereomicroscope (SMZ.168, Motic. Richmond, British Columbia, Canada), manually, using a razor blade. A group of the slices was used to obtain cuts in vibratome, as described next. The remaining slices were placed inside a tissue processor (Leica) to obtain tissue blocks embedded in paraffin.

The initial immunohistochemical study was performed in cuts of 50 µm thick obtained from a vibratome (VT1000-S, Leica Biosystems Nussloch GmbH, Heidelberger, Germany). These slices were collected in small Petri dishes (2.5 cm diameter) with PBS. During the entire procedure, the slices remained floating and in constant agitation at room temperature (20–22 °C, and with a humidity of 30%). Initially, the slices were treated with ammonium chloride (NH_4_Cl_2_) (Merck) 0.05 M for 30 min to remove aldehyde remnants, and with hydrogen peroxide (H_2_O_2_) (Merck) at 3% to inactivate endogenous peroxidase. To block nonspecific immunoreaction places, a solution composed by normal horse serum was used (Merck Millipore, Darmstadt, Germany) and bovine serum albumin (Sigma, St. Louis, MO, USA) and Triton X-100 (MP-Biomedicals, LLC, Fountain Parkway, USA). Then, the slices were incubated in the primary antibody for the detection of rabies antigens. To do so, an anti-rabies polyclonal antiserum (dilution 1:1500), elaborated and tested in our laboratory, was used [10]. Afterward, cuts were incubated in anti-rabbit secondary antibody (Sigma) (dilution 1:600), and then they were treated with ABC complex (Ref. SK4000, Vector Laboratory INC, Burlingame, CA, USA). The developing for the immunohistochemical detection of rabies antigens was performed with diaminobenzidine (DAB-Nickel, Ref. PK4100, Vector Laboratory INC). Details of these procedures have been described previously [10,17,18].

From the blocks embedded in paraffin, 7 µm thick sections were obtained in a conventional microtome (JUNG RM2045, Leica), and they were collected in glass slides. Some of these cuts were used to make conventional histological preparations colored with hematoxylin and eosin (H&E) (Sigma). The other cuts were destined for the immunohistochemical study of rabies. These were deparaffinized placing the slides containing them in a stove at 60 °C for the entire night. Then, they were treated with xylol (Merck) for 15 min, followed by absolute ethanol (J.T.Baker) for 15 min. The following steps of the immunohistochemical procedure were made inserting the slides in a humid chamber. The cuts were washed with PBS, treated with trypsin 0.1% for 30 min (Sigma), and then with EDTA 0.5% for 30 min (Sigma). After washing them with PBS, the cuts were treated with the blocking solution (described previously), followed by the incubations in anti-rabies primary antibody (dilution 1:800) and anti-rabbit secondary antibody (dilution 1:200), treated with ABC complex and stained with DAB. Finally, the cuts were contrasted with Harris’ hematoxylin (Merck) and the histological preparations were mounted with Entellan (Merck).

### 2.4. Electron Microscopy

To ease the penetration of the reagents in the nervous tissue and to maintain the orientation of the pyramidal neurons of the cerebral cortex, coronal slices of 150-µm thick at the level of the frontal cortex, upwards the striatum and the callous body, were obtained as anatomical referents. These slices were postfixed in osmium tetroxide (OsO_4_ Polysciences Inc., Valley Road Warrington, PA, USA) at 1% for 2 h, dehydrated in alcohols in increasing concentrations (50%, 70%, 90%, 95%, 100%) for 15 min in each of them, and then transferred to propylene oxide (OP)-(Polysciences) for 30 min (2 of 15 min). For imbibition and inclusion in epoxy resin Epon-Araldita (EA)-(Polysciences), the samples underwent the following sequence of mixtures of OP and EA (1:2, 1:1; 2:1) for 2 h in each of them. Then, the slices were placed in pure EA resin for the entire night. Finally, fragments of about 0.5 mm^2^ containing the entire length of the cerebral cortex were cut, and placed inside cylindrical capsules with flat bottoms. The capsules were filled in with fresh EA resin and polymerized for 48 h at 70 °C. In an ultramicrotome (Power Tome X, RMC products, for Boeckeler Instruments, Inc, Tucson, AZ, USA) semithin slices (500 nm) were obtained and colored with toluidine blue (Merck), to observe them in the optic microscope and select the specific areas for the electron microscopy. Then, ultrathin slices (60 nm) that were collected in grids and colored with uranyl acetate (Merck) and lead citrate (Merck) were obtained to observe them with an electron microscope (EM 109, Carl Zeiss Promenade, Jena, Germany).

### 2.5. Pre-Embedding Ultrastructural Immunocytochemistry

Some of the 150 µm thick slices obtained in the vibratome were subjected to the immunohistochemical reaction for rabies detection, and then they were processed for electron microscopy, following similar protocols to the ones described previously in this section, with some changes (Triton was not used, postfixation was made with OsO_4_ at 0.5% for 30 min and the resin used was Durcupan) (Sigma). This procedure, called “pre-embedding ultrastructural immunocytochemistry” or “pre-embedding immunoelectron microscopy” [19], is based on the reaction between DAB and OsO_4_, which forms an electro-dense granular precipitate that corresponds to the antigen distribution and is easily visible in the electron microscope.

## 3. Results

Macroscopic differences were observed between the brains of the mice fixed by perfusion and those fixed by immersion. The perfusion extracted completely the blood in the brains, and gave them a hard consistency facilitating their extraction from the skull and obtaining the slices. In the brains of the animals sacrificed by euthanasia and immersion-fixed, large amounts of blood in the tissue and a soft consistency was observed, the latter increased with the postmortem degradation time. This complicated the extraction and the execution of the cuts in the vibratome. The lack of firmness in the tissue affected the uniformity in the thickness of the slices. On the other side, the histomorphological changes described next, by the effect of postmortem degradation, were similar in the samples of the animals infected with the rabies virus and those of the controls. There was no evidence that the infection intensified postmortem degradation.

### 3.1. Hematoxylin and Eosin Staining

**Figure 1:**
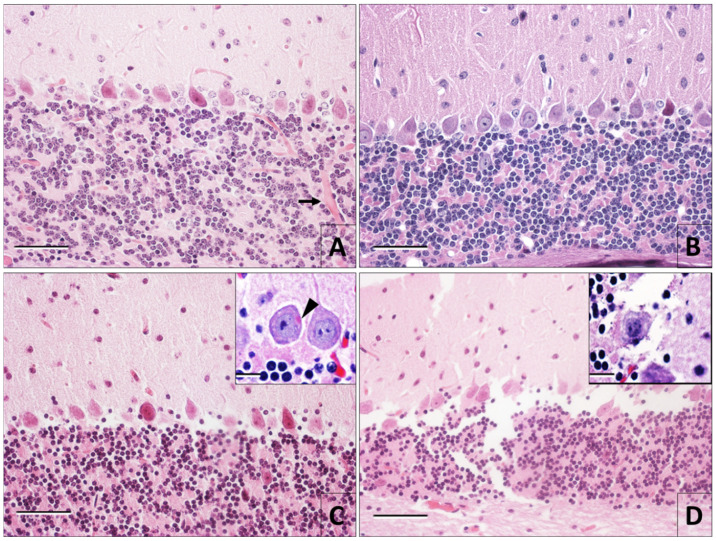
Hematoxylin and eosin stain in slices of cerebellum infected with rabies virus. Tissue fixed by immersion (**A**) and perfusion (**B**) at 0 h postmortem. Tissue integrity, delimitation of the cytoplasm and the neuronal nucleus, and part of the dendrites of the Purkinje cells in the perfusion-fixed samples are preserved. In the immersion-fixed tissue, blood is observed in the blood vessels (arrow). At 12 h postmortem (**C**), the tissue degradation process is clear; granulation of the neuropile does not allow the observation of dendrites. Dissolution of cytoplasm and neuronal nucleus in some Purkinje cells is also noted. Well-preserved Purkinje cells can be seen in the box, one of them with an eosinophilic inclusion in the cytoplasm (arrowhead). (**D**) Tissue with 30 h of postmortem degradation. Notice the loss of integrity and the autolysis in Purkinje cells with an absence of eosinophilic inclusions (Magnifications A–D 40×, boxes 80×) (bars 50 = 50 µm).

In brain tissue at 0 h postmortem, regardless of the fixation method, there was no sign of histomorphological changes associated with infection with the rabies virus. Effects of postmortem degradation were evident after hour six, where vascular dilatation in the tissue and the beginning of chromatin condensation were observed. The increase of postmortem time caused cytoplasmic degradation and granulation of the neuropile. However, these changes were not observed uniformly throughout the entire brain tissue. On the tissue infected with rabies, the presence of eosinophilic inclusions in the cytoplasm of the neurons was detected until 12 h postmortem. After 18 h postmortem, eosinophilic inclusions were not observed anymore, because of the loss of their eosinophilia and tissue degradation (Figure 1).

### 3.2. Immunohistochemistry of Rabies

**Figure 2:**
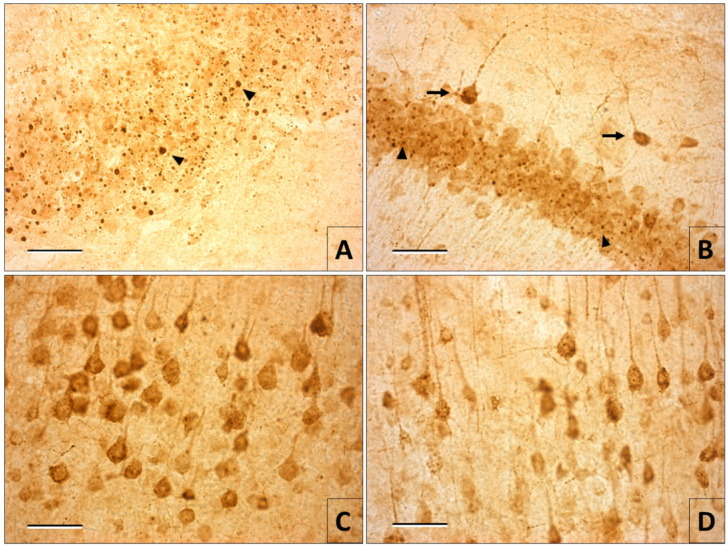
Immunohistochemistry for rabies in 50 µm thick slices from vibratome. (**A**) Perfusion-fixed cerebellum slice from an animal. Note the immunoreactivity of the intracytoplasmic viral inclusions (arrowheads), but there is no cytoplasmic marking that allows us to delimit the cellular morphology. (**B**) Cerebellum with 6 h of postmortem degradation, where several viral inclusions (arrowheads) and morphology delimitation of the Purkinje cells are observed (arrows). (**C**) Neurons of the cerebral cortex at 12 h postmortem. The immunostaining delimits the perikaryon morphology and a short segment of the apical dendrite, but it is hard to identify intracytoplasmic viral inclusions. (**D**) Cortical pyramidal neurons at 30 h postmortem. There is a loss of neurons in some areas due to degradation, but in others, the intense immunoreactivity of the soma and of the apical dendrite is preserved (Magnification A–D 40×) (bars = 50 µm).

**Figure 3:**
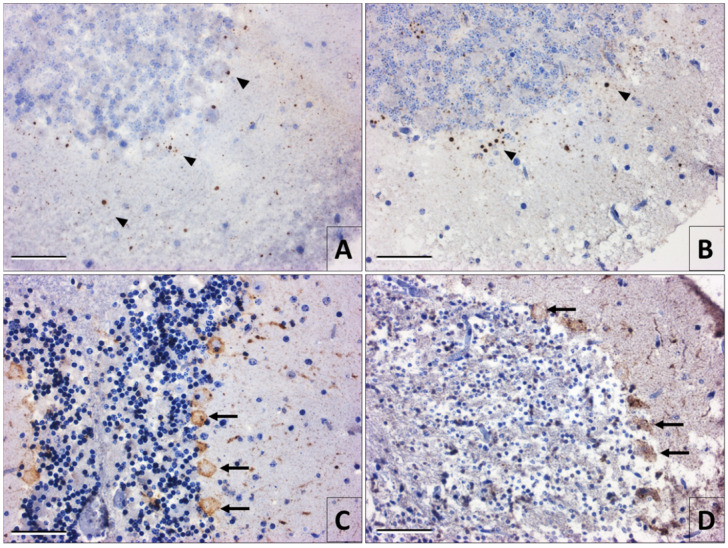
Immunohistochemistry for rabies in cerebellar slices (7 µm) embedded in paraffin. Tissue fixed by perfusion (**A**) and immersion (**B**) at 0 h postmortem. Many intracytoplasmic viral inclusions are observed in the area of the Purkinje cells (arrowheads), but the cellular morphology is not clear. In samples fixed at 12 h postmortem (**C**), immunostaining delimits the profile of the neuronal soma (arrows). At 30 h postmortem (**D**), the tissue presents degradation, but immunoreactivity is preserved in the soma (arrows) and some dendrite fragments of the Purkinje cells (Magnification A–D 40×) (bars = 50 µm).

The presence of viral antigens was revealed in every sample from the infected mice. In tissues fixed by perfusion or immersion, at 0 h postmortem, several intracytoplasmic viral inclusions well defined and scattered in all the layers of the cerebral cortex and the cerebellum were observed. However, immunoreactivity was not observed in the neuronal bodies. In samples with greater postmortem degradation times, the viral antigens were visible in the somas until the neuronal morphology and the fragments of dendrites were completely demarcated. This seems to mask the presence of intracytoplasmic viral inclusions in some neurons, although in others its absence is obvious (Figure 2 and Figure 3). This increase in the immunoreactivity was specific to the neurons in the samples of the animals infected with the virus; it was not observed in controls.

### 3.3. High-Resolution Optical Microscopy

**Figure 4:**
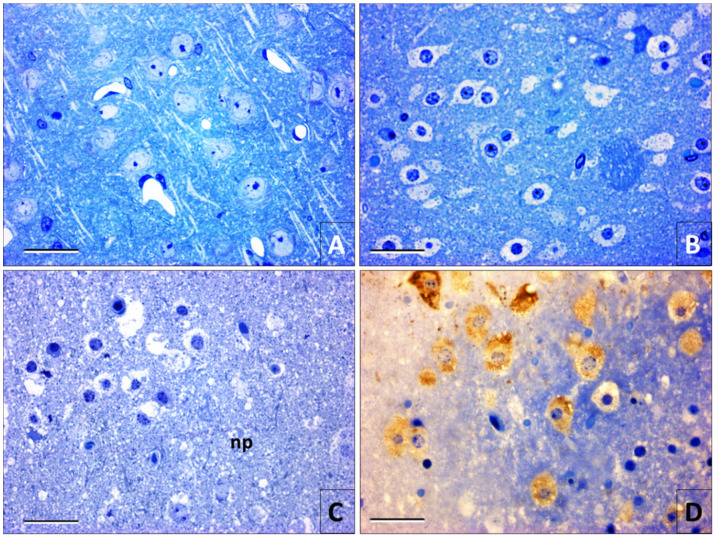
Images from semi-thin slices of mouse cerebral cortex in samples embedded in epoxy resins and stained with toluidine blue. (**A**) Tissue fixed at 0 h postmortem where normal cytomorphology and neuropile are observed. Neuronal nuclei are clear in appearance and the presence of one or more nucleoli is highlighted. (**B**) Sample fixed after 6 h of postmortem degradation. The autolysis, the chromatin condensation, and the nucleus retraction are evident, as well as the granulation of the neuropile. (**C**) Tissue fixed at 30 h postmortem where the granular neuropile (np) dominates, loss of neuronal morphology by autolysis and nuclei retraction. (**D**) Sample of material fixed at 30 h postmortem processed for pre-embedding immunomicroscopy in Durcupan. The viral antigens immunoreactivity delimits the profile of the soma in infected neurons despite the autolysis (Magnification A–D 40×) (bars = 50 µm).

From the tissue samples embedded in epoxy resins, 500 nm thick sections were obtained, and were then stained with toluidine blue for observation under the optical microscope. In the slices of the specimens taken at 0 h postmortem, apparent morphological changes were not observed (Figure 4A). Effects of tissue degradation were evident at 6 h postmortem when the beginning of autolysis, chromatin condensation, nucleus retraction, and granulation of the neuropile were observed (Figure 4B). These histological changes were more accentuated with a greater postmortem time. At 30 h, it was only distinguishable the retracted nucleus but not the profile of the perikaryon in most neurons (Figure 4C). In semithin slices of the samples processed for immunomicroscopy, fixed at 30 h postmortem, it was noticeable that the immunoreactivity of the viral antigens, including some intracytoplasmic viral inclusions, was occupying all the space of the neuronal cytoplasm (Figure 4D).

### 3.4. Electron Microscopy

**Figure 5:**
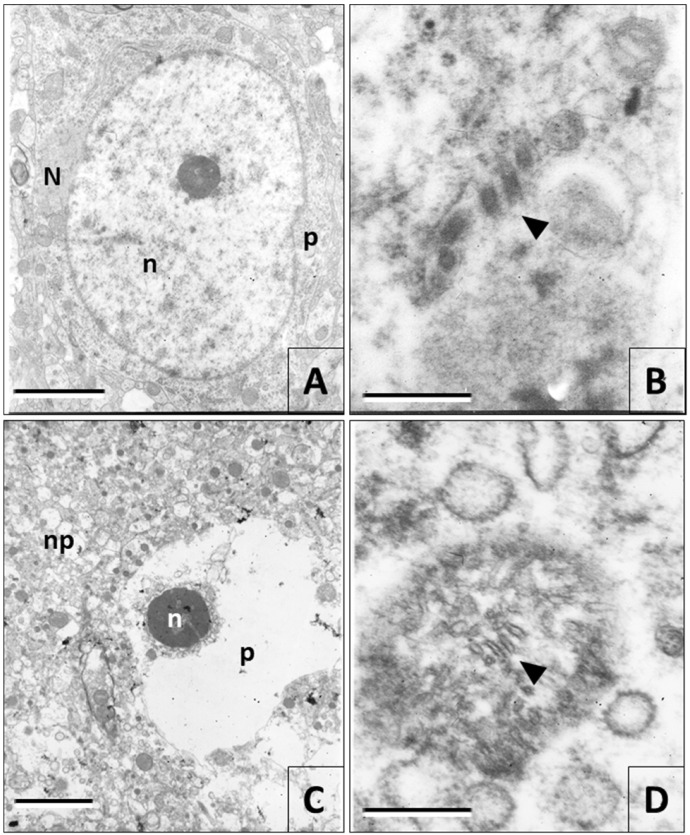
Electron microscopy images. (**A**) Picture of a neuron of a mouse cerebral cortex infected with rabies and fixed by perfusion (0 h postmortem). Alterations in the nucleus ultrastructure (*n*) or the perikaryon (*p*) were not observed, but the presence of a viral ribonucleoprotein inclusion (N) in the cytoplasm is noted. (**B**) Rabies virus particles (arrowheads) in brain tissue at 0 h postmortem. (C and D) Ultrastructural images of nervous tissue processed at 30 h postmortem. (**C**) Neuropile (np) degradation is noteworthy, as well as that of a neuron presenting nucleus retraction (n) and autolysis. (**D**) Structure that probably corresponds to a mitochondrion in a degradation process with fragments of membranes that can be confused with viral particles (arrowhead). (Magnifications. A = 7000×; B = 50,000×; C = 3000×; D = 50,000×) (Bars. A = 2400 nm, B = 200 nm, C = 4800 nm, D= 200 nm).

In the samples observed with the electron microscope, there was no evidence of ultrastructural changes in the tissue infected with the rabies virus fixed at 0 h postmortem (Figure 5A,B), except for the formation of viral ribonucleoprotein inclusions (Figure 5A). The first signs of ultrastructural damage in the brain tissue were noticed starting at 6 h postmortem. Nonetheless, tissue degradation did not occur uniformly in the tissue. Some areas were better preserved than others. As the postmortem degradation time increased, autolysis and nucleus retraction were the most characteristic features in neurons (Figure 5C), while the nuclei morphology was better preserved in the glial cells.

The changes in the ultrastructure of the intracytoplasmic organelles were noticeable, especially in mitochondria, which were gradually losing their membranous structure (Figure 5D). Viral particles were more visible in the electron microscope, but only in the samples fixed until 12 h postmortem. In general, the postmortem degradation observed in the electron microscope was similar in the samples inoculated with the rabies virus and their controls. The viral infection did not generate additional postmortem alterations.

**Figure 6:**
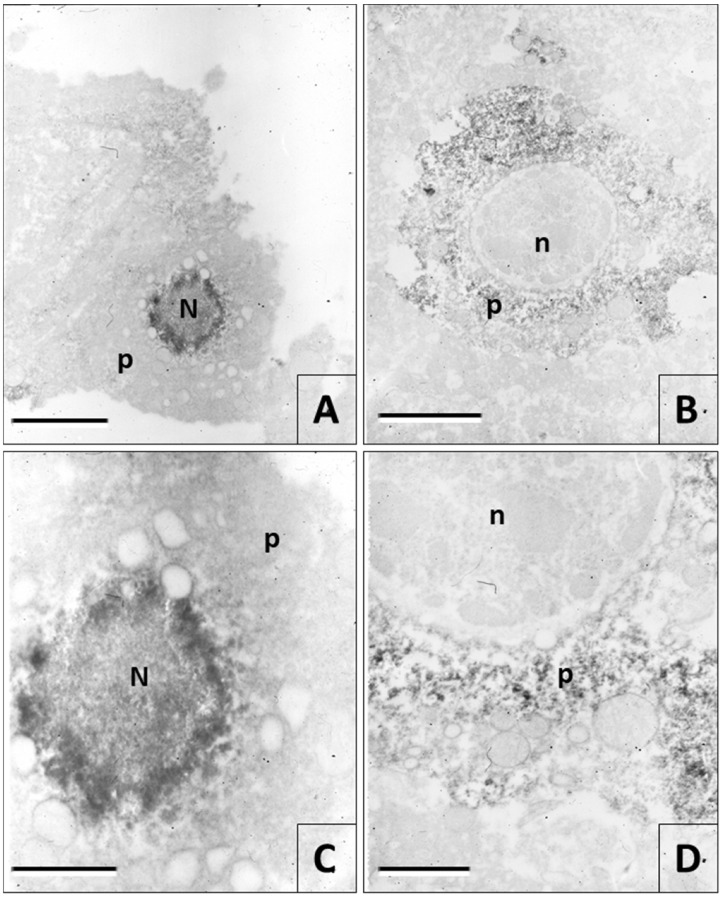
Immune electron microscopy images. (**A** and **C**) Intracytoplasmic viral inclusion or Negri body (N) from a sample fixed by immersion at 0 h postmortem. The electro-dense precipitate indicates a strong immunoreactivity in the periphery and masks the possible presence of viral particles. (**B** and **D**) Immunoreactivity to rabies in the neuronal perikaryon (*p*) at 24 h’ postmortem. The immunohistochemical reaction is scattered in the cytoplasm without the presence of Negri bodies. Rabies antigens are not observed in the nucleus (n). (Magnifications A = 7.000×; B = 4.400×; C = 30.000×; D = 20.000×) (Bars. A = 2400 nm, B = 4000 nm, C = 600 nm, D = 800 nm).

With the immune electron microscopy, it became evident the viral antigens distribution on the neuronal cytoplasm until 30 h postmortem, which was revealed by the reaction between the diaminobenzidine and the osmium tetroxide (Figure 6). Nevertheless, with this technique, intracytoplasmic viral inclusions or viral particles were not observed after 18 h postmortem (Figure 6B,D).

## 4. Discussion

The postmortem preservation of brain tissue is influenced by external factors, such as premortem and postmortem conditions and the conservation methods (fixation and freezing). Strict control of these variables is critical for good results in histochemistry and immunohistochemistry [20]. The processes of postmortem degradation can result in structural damages, changes in pH, cerebral edema, alterations of structural epitopes and in the chemical integrity of matrix components, and synapsis [5,21,22]. They can be a problem for the preservation of antigens in the diagnosis [5], as in the case of rabies.

Rabies postmortem diagnosis is made by means of laboratory tests, with nervous tissue extracted from skulls. In some cases, the tests are not performed, due to cultural or religious beliefs. In other cases, it is necessary to carry out the diagnosis with brain tissue subjected to fixation in aldehydes, especially when there are no signs that suggest rabies, or if there are problems with the transport of fresh samples. Besides, the diagnosis requires experience and expensive equipment, so they are usually carried out only in reference laboratory [4,6].

In this work, the brain tissue processed at 0 h postmortem did not suffer apparent morphological changes, and there was no record of cells in processes of cell death associated with infection with the rabies virus in any of the samples processed for H&E, immunohistochemistry, or electron microscopy. Usually, it is thought that rabies does not affect significantly the cytomorphology of the nervous tissue when it is studied with conventional methods [23]. Nonetheless, recent studies have highlighted the effect of the infection on the morphology of the dendrites [15,16,18,24,25].

It is believed that the rabies virus does not cause cell death by apoptosis in natural conditions [26,27]. Occasionally, neuronal pyknosis or necrosis occurs [23,28]. Experimentally, apoptosis can be induced with CVS viruses in suckling mice or recently weaned, especially when the inoculation is intracerebral [27,29].

The presence of eosinophilic inclusions in the cytoplasm is the only evidence of the infection in the brain tissue when it is processed by H&E. A diagnosis based only on the demonstration of the eosinophilic inclusions is not reliable, given that this structure is not always observed in the infected tissue [4,6]. Rabies virus inclusions are not clearly observed, or they are absent in between 20% and 60% of the samples infected with the virus, although immunohistochemistry improves their visibility [9,11]. As a result of this, it has been suggested that histological methods like the Seller and the H&E should not be recommended anymore, due to their very low sensitivity [30]. In our study, the eosinophilic inclusions were observed until 12 h postmortem, with both optical microscopy and electron microscopy. After this time, it was difficult to observe them, due to their disintegration within the tissue. Other authors have highlighted the inconvenience of diagnosing rabies by searching intracytoplasmic viral inclusions in tissues with advanced degradation processes, due to the probability of false negatives [31,32].

We accomplished the confirmation of the rabies virus infection in all the samples processed for immunohistochemistry until 30 h postmortem. What turned out to be surprising, but at the same time useful, was the increase of immunoreactivity to rabies antigens as the postmortem degradation time increased. This response was very obvious in the immunostaining revealed with diaminobenzidine for optical microscopy. The electron immune microscopy confirmed that this observation was not an artifact of the immunohistochemical technique. The increase of immunoreactivity with a greater postmortem time could be explained by a greater dispersion of the viral antigen after the disintegration of the intracytoplasmic viral inclusions associated with the effect of cytoplasmic degradation caused by cell autolysis.

On an immunohistochemical study of the glial fibrillar acidic protein (GFAP) in mice brains, the immunoreactivity increased significantly after 6 h postmortem. The authors suggest that this happened possibly because of the disintegration of the glial protein filaments, and the dispersion of their antigenic epitopes throughout the cytoplasm [5]. Nevertheless, there is a greater tendency to the decrease of the immunoreactivity due to postmortem degeneration in other antigens of the central nervous system [5,33].

A molecular test has been recently developed that can be suitable for the postmortem diagnosis of rabies [34]. The results have demonstrated the robustness of viral RNA, compared to viable virus or antigen in a decomposing animal, at least 70 days after death, even when the integrity of the brain tissue was compromised [6,35], which supports the use of a RT-PCR as a more sensitive and specific diagnostic test, since it can detect the rabies virus genome in samples highly decomposed [35,36,37], even when the Direct Fluorescent Antibody Test (DFA) and the Mouse Inoculation Test (MIT) present negative results [35]. However, there are financial and logistical barriers that prevent the routine use of molecular trials for the rabies diagnosis in many parts of the world [4].

In conclusion, immunohistochemistry is a reliable test for rabies virus diagnosis in material that has suffered some level of postmortem degradation, or that has been fixed with aldehydes. In addition, the use of fixation with paraformaldehyde in these kind of samples is recommended, because it attenuates the autolitical postmortem processes, preserves the natural morphology, and optimizes the macromolecules for the histochemical and biochemical analysis [38,39]. Future studies evaluating the effect of postmortem degradation in the detection of the rabies virus in brain tissue should include the direct rapid immunohistochemical test (dRIT). This has shown a sensitivity and specificity equivalent to those of the DFA. Additionally, the dRIT is simple, requires no specialized equipment or infrastructure, and can be successfully performed on samples preserved in glycerol solution for 15 months, formalin-fixed, or frozen for 24 months and in various conditions of preservation [12,13,40]
